# Essential Oil Composition and Antibacterial Studies of *Vitex negundo* Linn. Extracts

**DOI:** 10.4103/0250-474X.44610

**Published:** 2008

**Authors:** S. L. Khokra, O. Prakash, S. Jain, K. R. Aneja, Yogita Dhingra

**Affiliations:** University Institute of Pharmaceutical Sciences, Kurukshetra University, Kurukshetra-136 119, India; 1 Department of Pharmaceutical Sciences, Guru Jambheshwar University, Hisar-125 001, India; 2 Department of Microbiology, Kurukshetra University, Kurukshetra, Haryana-136 119, India

**Keywords:** *Vitex negundo*, essential oils, antibacterial studies, α-selinene, germacren-4-ol, carryophyllene oxide, δ-guaiene, carryophyllene epoxide

## Abstract

Essential oils from fresh leaves, flowers and dried fruits of *Vitex negundo* were obtained by hydrodistillation. Using Soxhlet extractor five successive extracts from dried and powdered leaves were also taken. The chemical constituents of essential oil of leaves, flowers and dried fruits were analyzed by GC-FID and GC/MS techniques. Main constituents identified in leaves oil were δ-guaiene, carryophyllene epoxide and ethyl-hexadecenoate; in flowers oil - α-selinene, germacren-4-ol, carryophyllene epoxide and (E)-nerolidol while fruit oil showed β-selinene, α-cedrene, germacrene D and hexadecanoic acid as the main constituents. β-Caryophyllene was only the constituent identified as common to all three oils. α-Guaiene and guaia-3,7-diene were identified as common constituents in leaf and dried fruit oil while leaf and flower oils showed *p* -cymene, valencene, caryophyllene epoxide and (E)-nerolidol as common constituent. All the essential oils and successive extracts were evaluated for antibacterial potential against *Staphylococcus aureus, Bacillus subtilis, Escherichia coli* and *Pseudomonas aeruginosa* bacterial strains. Each of the essential oils and extracts were found to give promising results against *B. subtilis* and *E. coli.* Ethyl acetate and ethanol extracts showed prominent antibacterial activity against all the tested strains. Fruits and leaves oil were found to be most active against *E. coli* and *S. aureus,* respectively. Only flowers oil was found to be active against *P. aeruginosa.*

*Vitex negundo* (family: Verbenaceae), is an important medicinal plant found throughout India. Though almost all of its parts are used in Ayurvedic and Unani systems of medicine, the extracts from its leaves and roots are the most important in the field of medicine and drug^1^. Its leaves^2^ and seeds^3^ are widely used externally for rheumatism and inflammations of joints and are also reported to have insecticidal properties. Internally, decoction of its leaves is taken as diuretic, expectorant, vermifuge, tonic and febrifuge^4^. The chemical components of the essential oil of leaf isolated from *V. negundo*^5^,^9^ and other *Vitex* species^10^ have been reported by several researchers in the past. Its essential oil is found to be useful for sloughing wounds and ulcers. The leaves of *V. negundo* are reported to possess pesticidal, antifungal and antibacterial properties^11^ so in the present study, besides GC/MS analysis of fresh leaves, flowers and dried fruits, the antibacterial potential of all three different oils and five successive extracts was studied. This study will be useful to identify the bioactive compounds of the oil, which may be responsible for the various medicinal properties of the plant.

The fresh leaves and flowers of *V. negundo* were collected in the month of August and September 2005 while fruits were collected in the month of October and November in the same year, from local areas of Kurukshetra, Haryana (India). The plant got identified and authenticated by FRI, Dehradun and a voucher specimen of the sample (Sr. No. 160/Flora of Haryana) has deposited in the NWFP Herbarium collection at Forest Research Institute and College, Dehradun, India.

The freshly collected leaves, flowers and dried fruits of *V. negundo* were washed twice with water to remove dust, just before hydrodistillation. Each of the leaves, flowers and fruits were then subjected to hydrodistillation separately for 8 h using a closed type Clevenger apparatus for extraction of oils lighter than water. Yellowish oil so obtained was separated from the distillate with the help of hexane and dried over anhydrous MgSO_4_. Percentage yield of essential oil was calculated and it was stored in sealed glass bottles in a refrigerator until analysis. In case of leaves oil extraction, before subjecting to hydrodistillation, the leaves were treated with a 10% KOH solution for 3 h and then washed twice with fresh water.

Shade dried leaves were pulverized and extracted successively with petroleum ether + ethyl acetate + CH_3_OH + C_2_H_5_OH + H_2_O in a Soxhlet extractor for 18 h. The solvents were evaporated under vacuum and percentage yield of each extract was calculated. Each of the extract was stored in a sealed glass bottle in a refrigerator until analysis.

GC analysis was carried out on Shimadzu GC17A GC V3 system, equipped with FID and fitted with a column (30 m × 0.25 mm, film thickness 0.2 μm). Temperature parameters: column oven- 100°, injection port- 250°, detector- 300°. Time programming: 100° for 2 min, temperature raised from 20° to 300° for 10 min, total time 22 min. Carrier gas used was He at 600 kPa primary pressure with 3 ml/min purge flow, column pressure 100 kPa.

GC/MS analysis was performed on Shimadzu GC/MS QP 5000 with GC17A GC system fitted with DB-6 chrompack capillary column (30 m x 0.25 mm, film thickness 0.2 μm). Temperature programming: 50° - 300° at 20°/min. Carrier gas used was He at 55 kPa pressure, 53 ml/min flow rate. Electron impact mode of ionization with ionization energy 70 eV and ion source temperature 170°. Peaks were identified by comparison of relative GC retention times with standards from literature, retention indices on BP - 1 column^12^–^13^, peak enrichment on co-injection with authentic standard wherever possible and comparison of mass spectra with literature data^14^–^16^. Relative percentage amounts were computed from GC peak areas without FID response factor correction.

The bacteria used for antibacterial tests were Gram (+) *Staphylococcus aureus* (MTCC 3160) *, Bacillus subtilis* (MTCC 0121) and Gram (−) *Escherichia coli* (MTCC 0051) *, Pseudomonas aeruginosa* (MTCC 0741). All the strains used for these studies were procured from MTCC, IMTECH, Chandigarh, India. Antibacterial potential of all three samples of essential oils and successive extracts was evaluated by agar well diffusion method. Nutrient agar plates were swabbed with the broth culture of the respective microorganisms (diluted to 0.5 McFarland Standard) and were kept at room temperature for 15 min for absorption to take place. Wells of 8 mm diameter were punched into the agar medium and filled with 100 μl each of the essential oils and extracts. DMSO, DMF and hexane were taken as solvent blank and Ciprofloxacin was used as positive control. The inoculated agar plates were incubated for 24 h at 37°. All the tests were made in triplicate and diameter of the inhibition zones was calculated in mm. The average of diameter of the inhibition zones of each sample was taken called clearing zone (CZ) and the antimicrobial index (AI) was computed as the clearing zone (CZ) minus the diameter of the hole divided by the diameter of the hole.

Out of thirty six constituents shown in GC spectra of leaf essential oil, only twenty constituents were identified. The identified constituent- *p*-cymene, *cis*-ocimene, citronellal, β-curcumene, β-caryophyllene, α-guaiene, guaia-3,7-diene, δ-guaiene, valencene, caryophyllene epoxide, ethyl-9–hexadecenoate, palmitic acid, (E)-nerolidol, humulene epoxide 1, globulol, humulene epoxide 2, epi-α-cadinol, α-muurolol, α-cadinol and α-bisabolol acetate represented about 85.5% of total composition of the essential oil of leaf. Out of twenty three constituent, twelve identified constituent in flower essential oil were formic acid, n-heptane, *p*-cymene, β-caryophyllene, trans-α-bergamotene, valencene, α-selinene, β-selinene, germacren-4-ol, caryophyllene epoxide, (E)-nerolidol and *P*-(1,1-dimethylethyl) toluene represented about 65% of total composition of the oil. The thirteen constituents namely α-copaene, β-caryophyllene, α-cedrene, α-guaiene, guaia-3,7-diene, α-humulene, aristolene, germacrene D, β-selinene, caryophyllene oxide, n-hexadecanoic acid, palmitolic acid and traces of acetyl lactyl glycerate were identified in dried fruit oil out of seventeen. Identification of aristolene, n-hexadecanoic acid, palmitolic acid and acetyl lactyl glycerate was tentative. The chemical compositions of the essential oil have been presented in Table 1. In dried fruit oil three major constituents β-selinene, α-cedrene and germacrene D were representing approximately 50% (by GC peak area) of the total composition. The constituents α-cedrene, guaia-3,7-diene, palmitolic acid and acetyl lactyl glycerate were being reported for the first times in oils of *V. negundo*. Ethyl-9-hexadecenoate, caryophyllene epoxide, δ-guaiene, β-caryophyllene and palmitic acid were the most abundant constituents in leaf essential oil while α-selinene, caryophyllene oxide, germacren-4-ol, (E)-nerolidol, β-caryophyllene and n-heptane were the major constituents of flower essential oil. β-Caryophyllene was only the constituent identified as common to all three oils in the same proportion. α-Guaiene and guaia-3,7-diene were identified as common constituents to leaf and dried fruit oil while leaf and flower oils showed *p*-cymene, valencene, caryophyllene epoxide and (E)-nerolidol as common constituent. An earlier analysis of essential oil of *V. negundo* leaf^6^,^9^ indicated presence of β-caryophyllene, caryophyllene oxide, globulol, sabinene, and viridiflorol as major constituents. The results of present investigation matches with the results of earlier findings except in that one constituent namely viridiflorol was not found in these studies. The major constituents named ethyl-9-hexadecenoate, δ-guaiene, caryophyllene epoxide, valencene, α-selinene and germacren-4-ol were found first time along with thirteen minor constituents in essential oil of leaf of *V. negundo*. Limonene, 1,8-cineole, citral, cinnamic aldehyde, eugenol, terpinen-4-ol^5^,^10^ sabinene and viridiflorol^6^,^9^, which were reported as major constituents of the oil of leaves could not be detected in the present studies. Fig. 1 shows the results of well diffusion test and zone of inhibition of tested samples against blank. The blank solvents DMSO, DMF and hexane did not show any zone of inhibition. All the extracts and essential oils were found to be highly effective in inhibiting the growth of bacteria at a minimum concentration of 30 and 60 μg/100 μl, respectively (Table 2). Each of the essential oil and extracts were found to be active against *B. subtilis* and *E. coli* with antimicrobial index (AI) ranging from 0.3 to 1.8. Leaf essential oil inhibited *S. aureus* with maximum AI of 1.5 while fruit essential oil showed its inhibition against *E. coli* and *B. subtilis* with AI of 1.3 and 1.0, respectively. Flower oil did not show any activity against *S. aureus* while leaf and fruit oils were ineffective against *P. aeruginosa*. Ethyl acetate extract was found to be most potent among all the extracts tested. Petroleum ether and aqueous extracts did not show any activity against *P. aeruginosa* while all the extracts were found potent against *S. aureus*. Ciprofloxacin was used as positive standard control and the results of tested samples were very promising in comparision to standard drug ciprofloxacin.

**TABLE 1:** CHEMICAL COMPOSITION OF LEAVES, FLOWER AND DRIED FRUIT ESSENTIAL OIL OF *VITEX NEGUNDO*

Leaf oil Constituents	Retention index	Peak Area (%)	Flower oil Constituents	Retention index	Peak Area (%)	Dried Fruit oil Constituents	Retention index	Peak Area (%)
*p*-cymene	1006	0.8	formic acid*	---	0.7	α-copaene	1364	5.4
*cis*-ocimene	1027	3.3	*n*-heptane*	---	5.2	β-caryophyllene	1403	5.0
citronellal	1126	2.3	*p*-cymene	1009	0.1	α-cedrene*	1409	14.0
β-curcumene*	---	0.3	β-caryophyllene	1403	5.2	α-guaiene	1419	1.5
β-caryophyllene	1403	5.0	trans-α-bergamotene*	1436	0.2	guaia-3,7-diene*	1430	2.0
α-guaiene	1420	1.3	valencene*	1471	2.4	α-humulene	1434	4.0
guaia-3,7-diene*	1430	0.9	α-selinene*	1470	17.0	Aristolene	1455	8.0
δ- guaiene*	---	18.0	β-selinene	1473	0.3	germacrene D	1459	8.0
valencene*	1471	2.3	germacren-4-ol*	1530	9.0	β-selinene	1464	22.0
caryophyllene epoxide*	1550	10.2	caryophyllene epoxide	1550	15.2	caryophyllene oxide	1555	3.0
ethyl-9-hexadecenoate*	---	28.5	(E)-nerolidol	1552	8.0	*n*-hexadecanoic acid	------	8.0
palmitic acid*	---	5.2	*p*-(1,1-dimethylethyl) toluene*	---	1.2	palmitoleic acid	----	3.0
(E)-nerolidol	1552	3.0				acetyl-lactylglycerate	----	1.5
humulene epoxide 1*	1575	1.0						
globulol	1585	0.8						
humulene epoxide 2*	1608	t						
epi-α-cadinol*	1640	0.7						
α-murolol*	1648	1.0						
α-cadinol	1653	t						
α-bisabolol acetate*	1798	0.8						

t - trace (>0.1%)

* Newly reported in this oil

**Fig. 1:**
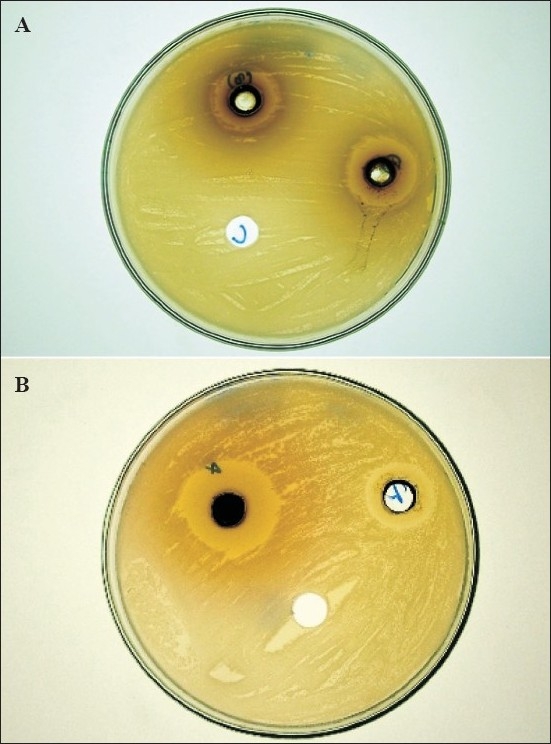
Antibacterial activity of *Vitex negundo* Linn. extracts (A) Againt *Pseudomonas aeruginosa*, 5-ethylacetate extract (AI=1.4), 7-ethanolic extract (AI=1.2), C-DMSO (AI=0) and (B) against *Escherichia* coli 2-flowers oil (AI=0.8), 4-petroleum. ether extract (AI=1.8), C-DMF (AI=0)

**TABLE 2:** ANTIBACTERIAL ACTIVITY OF ESSENTIAL OILS AND SUCCESSIVE EXTRACTS OF *VITEX NEGUNDO*

Extracts/Essential oils	Concentrations (μg/100μl) of diluents	*Staphylococcus aureus*	*Bacillus subtilis*	*Pseudomonas aeruginosa*	*Escherichia coli*
					
		CZ	AI	CZ	AI	CZ	AI	CZ	AI
Leaves oil	60 / hexane	20.3	1.5	16.0	1.0	---	---	15.0	0.8
Flower oil	60 / hexane	---	---	11.0	0.3	13.0	0.6	14.3	0.8
Fruit oil	60 / hexane	14.6	0.8	16.3	1.0	---	---	18.3	1.3
Pet. ether extract	30 / DMF	28.0	2.5	20.8	1.6	---	---	22.4	1.8
Ethyl acetate extract	30 / DMF	33.6	3.2	22.4	1.8	19.2	1.4	20.8	1.6
Methanol extract	30 / DMSO	19.2	1.4	15.2	0.9	14.4	0.8	15.2	0.9
Ethanol extract	30 / DMSO	28.0	2.5	16.0	1.0	17.3	1.2	14.6	0.8
Aqueous extract	30 / DW	10.4	0.3	19.2	1.4	---	---	12.8	0.6
Ciprofloxacin	10/DMSO	25.0	2.2	27.0	2.3	28.0	2.5	27.0	2.3
DMF	pure	---	---	---	---	---	---	---	---
DMSO	pure	---	---	---	---	---	---	---	--
Hexane	pure	---	---	---	---	---	---	---	---

---: no inhibition zone, DMF: dimethyl formamide, DMSO: dimethylsulfoxide, DW: distilled water, AI- Antimicrobial index, CZ - Clearing zone (mm). The average of diameter (mm) of the inhibition zones called clearing zone (CZ) and the antimicrobial index (AI) was computed as the clearing zone (CZ) minus the diameter of the hole divided by the diameter of the hole.
